# Effectiveness of sensory modulation for people with schizophrenia: A multisite quantitative prospective cohort study

**DOI:** 10.1111/1440-1630.12803

**Published:** 2022-04-19

**Authors:** Tawanda Machingura, David Shum, Chris Lloyd, Karen Murphy, Evelyne Rathbone, Heather Green

**Affiliations:** ^1^ Faculty of Health Sciences & Medicine Bond University Gold Coast Queensland Australia; ^2^ School of Applied Psychology Griffith University Gold Coast Australia; ^3^ Hong Kong Polytechnic University Hong Kong Hong Kong

**Keywords:** mental health, occupational therapy, psychosocial intervention, quantitative evaluation, schizophrenia, sensory disorders

## Abstract

**Introduction:**

Current research evidence suggests that people with schizophrenia have sensory processing difficulties. Sensory modulation has growing evidence for use in this population. This study aimed to evaluate the extent to which health, social, cognitive, and occupational functioning outcomes were impacted by sensory modulation interventions for people with schizophrenia.

**Methods:**

A prospective observational cohort study using a waitlist control design was used in two large hospital and health services in Queensland, Australia. The study recruited patients who used sensory modulation (*n* = 30) across the two hospitals and those who did not use sensory modulation interventions as a control (*n* = 11). Results were analysed using a series of planned comparisons including independent and paired *t‐*tests, and mixed ANOVA was used whenever statistically indicated. The analysed measures were pre‐ and post‐intervention scores.

**Results:**

This study found no statically significant differences between the control and intervention groups at both pre‐ and post‐intervention. However, analysis of results from within the intervention group showed statistically significant improvements between pre‐ and post‐test scores on distress, occupational functioning, and health and social functioning but not on sensory processing and global cognitive processing. Further analysis of results from this study, compared with those from an earlier study on the general population showed significant differences in Low Registration and Sensation Avoiding, as measured by the Adult/Adolescent Sensory Profile, between participants with schizophrenia and those without schizophrenia.

**Conclusion:**

This study provides evidence to suggest that sensory modulation interventions can be complementary to standard care when utilised appropriately in clinical settings. Findings also suggest that the sensory profile of people with schizophrenia is different to that of the general population and this may have clinical implications. Further longitudinal research is needed with larger and randomised samples, using more targeted measures to better explore effectiveness of sensory modulation interventions.

**Key Points for Occupational Therapy**

- Sensory modulation interventions may be effective for providing distress relief and improving satisfaction with daily activities.
- People with schizophrenia may process sensory information differently from people without schizophrenia; this should be considered when providing services.
- A cautious approach needs to be adopted to match the state of the evidence.

## 1 INTRODUCTION

Schizophrenia is a complex mental illness that affects around 1% of the world's population, and individuals who experience schizophrenia have reduced life expectancy in the range of 10–25 years (Rahman & Lauriello, 2016). Despite being a debilitating illness, effective treatments for schizophrenia are limited to antipsychotic medication (Rahman & Lauriello, 2016) and a few psychosocial interventions with moderate effectiveness such as supported employment and social skills training (McDonagh et al., 2020). The problem with antipsychotic medications however is that they do not work for everyone and most people who take them experience side effects and some will consequently stop taking them (Cooper et al., 2020). The aim of this study was to explore the effectiveness of sensory modulation (SM), an emerging intervention already in use in clinical settings for people with schizophrenia.

### 1.1 Sensory modulation

People with schizophrenia have been reported to experience high rates of SM deficits when compared with the general population (Brown et al., 2002; Brown et al., 2020), and these sensory processing problems impact on daily occupational and social functioning (Barbic et al., 2019; Champagne, 2011a; Champagne et al., 2015; Fleischhacker et al., 2014; Lipskaya‐Velikovsky et al., 2015; Yakov et al., 2018).

Research into sensory processing patterns suggest that individuals with schizophrenia show higher scores on sensation avoiding and low registration, and lower scores on sensation seeking, than individuals with no psychiatric conditions (Brown et al., 2002). Several studies have concluded that interventions that target sensory processing, such as SM interventions, can improve occupational functioning in daily living activities (Barbic et al., 2019; Lipskaya‐Velikovsky et al., 2015; Sutton et al., 2013). An improvement in occupational functioning implies that individuals can more ably engage in purposeful and meaningful activities of daily life. These previous studies provide a basis for the use of SM interventions in practice.

SM intervention is an emerging practice used by health professionals, where sensory input is purposefully manipulated to help individuals optimally regulate and organise their responses to sensory input (Bar‐Shalita & Cermak, 2016; Champagne et al., 2010; Lipskaya‐Velikovsky et al., 2015). In occupational therapy practice, the purpose is to enhance effective participation in activities of daily living (Brown et al., 2019; Lipskaya‐Velikovsky et al., 2015). The practice involves giving specific types and amounts of sensation, at specific times, tailored to each individual, for therapeutic purposes (Champagne et al., 2010). This manipulation of sensory input is achieved using activities, behavioural strategies, specific equipment, and modification of the physical and social environment to assist the regulation of an individual's sensory experience (Sutton & Nicholson, 2011). The use of SM interventions is an emerging practice in mental health driven by contemporary approaches such as the recovery approach, trauma informed care, and seclusion and restraint reduction (Champagne, 2011a; Lloyd et al., 2014).

### 1.2 Previous studies on sensory modulation intervention effectiveness

A recent retrospective study on sensory processing patterns of people with a psychiatric condition concluded that those with a mental illness differ in sensory processing patterns to the general population and recommended condition‐specific sensory‐based interventions (Brown et al., 2020). Effective SM practice increases service users' awareness of their sensory preferences and assists them to manage their arousal through the application of sensory strategies (Sutton et al., 2013). Several scoping reviews and systematic literature reviews have concluded that there is limited evidence on the effectiveness of SM interventions (Machingura et al., 2018; Scanlan & Novak, 2015). Most studies are on general SM programmes with only a few looking at specific SM interventions.

A commonly reported SM intervention is the use of a specifically designed sensory environment in inpatient mental health units often called a “sensory room” or “chill out room” or “comfort room” (Lloyd et al., 2014; Yakov et al., 2018). Previous studies found that such sensory spaces or rooms had positive effects for users and staff. The benefits include practical and alternative opportunities for consumer de‐escalation, skill development, and increasing self‐awareness (Champagne et al., 2010; Lloyd et al., 2014; Sutton & Nicholson, 2011; Yakov et al., 2018). Sensory room data from these previous studies also showed a significant reduction in distress for consumers using SM interventions, which correlated with a reduction in seclusion and restraint occasions for those patients using SM interventions (Champagne et al., 2010; Lloyd et al., 2014; Sutton & Nicholson, 2011; Yakov et al., 2018).

Deep pressure stimulation using weighted modalities is another commonly used SM intervention reported in previous studies (Champagne et al., 2010; Yakov et al., 2018). Research studies on the therapeutic effectiveness of weighted blankets have demonstrated moderate effectiveness in the general population. In Mullen et al.'s (2008) concurrent, nested, mixed methods exploratory study on the therapeutic effects of weighted blankets, they found that 63% of participants had lower anxiety when using weighted blankets. Another exploratory study in an inpatient mental health unit found that 60% of participants reported a significant reduction in anxiety when they used weighted blankets (Champagne et al., 2015). However, the generalisability of these previous studies is limited because they had methodological problems such as relatively small samples, used convenience sampling, the samples being heterogenous, and not including control participants. Despite these limitations, the studies provided useful insights into the effectiveness of deep pressure stimulation.

Previous studies suggest that there are three main criteria for SM interventions to be effective for an individual: (1) The individual needs to develop some level of self‐awareness, (2) the individual needs to know what their sensory preferences are, and (3) the individual needs to integrate their sensory preferences into their daily life (Champagne, 2011b). Despite encouraging practice‐based evidence on SM interventions, studies with higher levels of evidence and less bias are needed to improve generalisability of this practice.

### 1.3 Current study

The problem of limited well‐designed research evidence to support SM interventions does not necessarily mean that interventions are not effective but rather that there are gaps in knowledge on the subject. This study therefore used standardised tools to report on and measure effectiveness in a relatively homogenous study population to mitigate the limitations reported in previous studies. Another important issue is that not all clinically important outcomes have been addressed in previous studies. Most looked at distress tolerance but did not address functional outcomes (Champagne, 2011a; Lloyd et al., 2014; Sutton et al., 2013; Yakov et al., 2018). This study sought to measure and report on both levels of distress and functional outcomes to bridge this gap.

The aims of this study were to determine how SM interventions impact people with schizophrenia and to establish whether people with schizophrenia had atypical sensory processing when compared with the general population from in the same geographic region. It was hypothesised that SM interventions would lead to greater improvements in reported occupational, health, and social outcomes for people with schizophrenia than treatment approaches that did not include SM interventions.

## 2 METHODS

### 2.1 Participants and recruitment procedures

This study was designed and proposed as a randomised controlled trial; however, the health ethics committee disallowed this design at the time due to potentially disadvantaging other patients from receiving what was regarded as a potentially beneficial intervention at the time. Subsequently, the research design was changed to a waitlist control design, which received ethical clearance from Gold Coast University Hospital Human Ethics Review Committee (HREC/17/QGC/151) and Griffith University Human Research Ethics Committee (GU: 2019/079). The study setting was two major hospital and health services in Southeast Queensland, Australia.

A prospective observational cohort study design with a waitlist control group was used. On one of the sites, participants were recruited from the Statewide Forensic Hospital within a High Secure Unit, a Medium Secure Unit, and an Extended Treatment Unit. The other site was a large hospital and health service with participants being recruited from three Psychiatric Acute Units and an Extended Treatment Psychiatric Unit. All units had a bed capacity of 16–24 patients, and care was delivered by a multidisciplinary team including occupational therapists, psychologists, social workers, nurses, and doctors.

Participants' diagnosis had to be schizophrenia or any one of its subtypes as diagnosed by a medical practitioner or psychiatrist based on the criteria of the Diagnostic and Statistical Manual of Mental Disorders, Fifth Edition (American Psychiatric Association, 2013). All participants had no previous experience of SM. This study had a total of 41 participants with schizophrenia, with 11 consumer participants in the control group and 30 consumer participants in the intervention group. The control group were those participants who had given consent to participate in the study but were prevented from doing so for practical reasons, which included unavailability of trained staff to provide the intervention and consumer moving to a ward where intervention was not being provided at the time.

### 2.2 Measures

Measures were administered at start (T0) and at 3 months (T1) and then at end (T2). At T0, both groups provided baseline measurements. The intervention group then received SM, whereas the control group continued with standard treatment including medications and psychosocial interventions. Measures were then collected again at T1 and at T2. Demographic details including age, sex, ethnicity, diagnosis, and phase of treatment were collected at the start. Phases of care are descriptions used by hospitals in Australia to describe the primary goal of care at a point in time and include Acute (short‐term reduction in severity of symptoms and/or personal distress); Functional (improvement of personal, social, or occupational functioning); Intensive extended (prevention or minimisation of further deterioration and risk reduction); Consolidating gain (maintain or improve the level of functioning during a period of recovery); and Assessment only, where the goal is to obtain information in order to determine the intervention or treatment needs (Independent Hospital Pricing Authority, 2016). The other measures collected for most participants at T0 and T2 as they got discharged from hospital.

#### 2.2.1 Occupational functioning

The Canadian Occupational Performance Measure (COPM) is a semi‐structured interview that enables an open dialogue between client and therapist on issues of importance to the client (Law et al., 1994). COPM is designed to detect a change in individual self‐perception of occupational performance over time in all areas of life, including self‐care, leisure, and productivity (Law et al., 1994).

The COPM has two main scores “Performance” and “Satisfaction”, each out of 10, where 1 indicates poor performance and low satisfaction, respectively, and 10 indicates very good performance and high satisfaction (Law et al., 1994). Mean “Performance” and “Satisfaction” scores are calculated, with higher scores indicating better occupational functioning. Internal consistency reliability, test–retest reliability, and validity of the COPM as a measure of occupational performance have been shown to be reasonable to good (Tuntland et al., 2016).

#### 2.2.2 Cognitive functioning

The Allen Cognitive Level Screen (ACLS) is a standardised cognitive screening tool. It uses an ordinal hierarchy of six distinct patterns of performance or cognitive levels from Level 1 to Level 6 (Allen et al., 2007). It is used to screen an individual's global cognitive processing abilities and is used as a guide to help determine an individual's optimal ability to function. This is determined by considering the task and environmental demands in relation to the cognitive abilities of the client. Level 1 indicates severe cognitive global impairment, whereas Level 6 indicates no global cognitive impairment. The inter‐rater reliability of the ACLS is high to very high with reported correlations ranging from *r* = .91 to *r* = .99, and there is a strong body of research supporting content, construct, and concurrent validity of the ACLS (Scanlan & Still, 2013).

#### 2.2.3 Distress

The Emotions Rating Scale (ERS) is a consumer self‐rating tool on a 10‐point scale (Champagne, 2011b). This is a non‐standardised tool whereby consumers self‐rate their emotions pre‐ and post‐SM interventions on a scale of 1 (*severe distress or tense*) to 10 (*calm or relaxed*). This scale is a two‐sided form, one labelled “before” and one labelled “after”. This scale was already in practical use by both patients and staff before and after each SM intervention session. Average before and after scores recorded for each client by the consumer themselves and by staff were later evaluated. Although not validated, this scale is commonly used by clinicians and researchers (Champagne, 2011b; Lloyd et al., 2014). People who did not receive SM interventions did not complete the ERS; therefore, ERS scores were only analysed in the treatment group.

#### 2.2.4 Health and social functioning

The Health of the Nation Outcome Scale (HoNOS) is mandated for use by all specialist mental health services in Australia. The HoNOS is a clinician‐rated tool used to measure the health and social functioning of adults 18–65 years using services. HoNOS is a set of 12 items that measure behaviour, impairment, symptoms, and social functioning. Each item is rated between 0 = *no problem* to 4 = *severe problem* or 7 = *not applicable* (Wing et al., 1998). In the data cleaning process, the ratings of 7 were treated as missing rather than being included in the analysis as raw scores.

HoNOS has been reported to have a moderately high internal consistency (Cronbach's alpha .59–.76); low item redundancy; and an adequate or good validity, reliability, sensitivity to change, and utility (Pirkis et al., 2005). Some studies have shown HoNOS to have good inter‐rater reliability and validity and sensitivity to therapeutic change (Wing et al., 1998), although others have reported low inter‐rater reliability in some circumstances (Green et al., 2007). Green et al. (2007) used HoNOS aggregate scores for the 12 items and found that these aggregate scores were consistent with the standardised Depression Anxiety Stress Scales (DASS)‐21. The present study also used HoNOS aggregate scores, for the two scales most relevant to the present study. These were Overactive, aggressive, disruptive or agitated behaviour and Problems with activities of daily living. Current research evidence suggests that SM interventions impact on behaviour and daily functioning (Lipskaya‐Velikovsky et al., 2015; Lloyd et al., 2014). A lower aggregate score indicates better health and social functioning.

#### 2.2.5 Sensory processing

The Adult/Adolescent Sensory Profile (A/ASP) is a 60‐item self‐report measure that measures frequency of responses to specific sensations (Brown et al., 2001). Respondents reflect on their everyday sensory experiences by indicating how often they respond to sensory experiences using a 5‐point scale from 1 = *almost never* to 5 = *almost always*. Each item corresponds to one of four quadrants: Low Registration, Sensation Seeking, Sensory Sensitivity, and Sensation Avoiding, reflecting different sensory processing (Brown et al., 2001). Scores that fall within one standard deviation of the mean for each category represent “Typical Performance”. Scores that fall between one and two standard deviations from the mean indicate “Probable Difference”, and scores that are more than two standard deviations from the mean indicate “Definite Difference”.

The A/ASP has fair internal consistency for ages 18 and above. For ages 18–64 years, the coefficient alpha values are .69 for Low Registration, .64 Sensation Seeking, .66 Sensory Sensitivity, and .70 Sensation Avoiding (Brown et al., 2002). Validity has been established using physiological studies, and support for construct validity of the measure has been established (Brown et al., 2001).

### 2.3 Intervention processes

**FIGURE 1:**
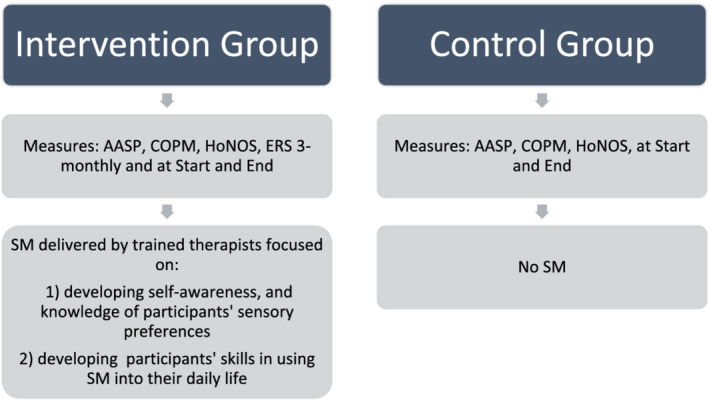
Intervention processes

SM interventions were delivered by registered occupational therapists who had received in‐service training on SM. SM training was delivered in a blended fashion including completion of the sensory approaches e‐learning training package developed by the Queensland Centre for Mental Health Learning (QCML) and the Occupational Therapy Mental Health Sensory Approaches Clinical Collaborative in Queensland (Meredith, Yeates, et al., 2018). This was then followed ongoing professional education on SM interventions with the first author. SM intervention training was initially developed by Champagne (2011b) and has been adapted for local relevance by QCML and local occupational therapists. Evaluation of QCML's e‐learning training package was found to lead to improvements in knowledge, confidence, and attitudes about sensory approaches soon after training; however, improvements were found to be decreased slightly after 3 months from training (Meredith, Hutchens, et al., 2018; Meredith, Yeates, et al., 2018). Sensory interventions followed a process where participants received SM Awareness training individually or in groups (approximately an hour) and then followed a specific daily programme developed with the individual participant by the occupational therapist and implemented over the course of the study. Fidelity of the interventions was moderated via standardised training for staff designed by QCML. The participants receiving the interventions were then followed and received repeat measures at 3‐monthly intervals up for period of up to 6 months as shown in Figure 1.

### 2.4 Procedures

Participants were recruited from the two sites described above, following hospital and university ethics approval. Recruitment was conducted by occupational therapists who were already treating clinicians to the participants at the two sites. These occupational therapists whose role was to provide SM and collected the data were not told the hypothesis of the study. Participants provided individual written informed consent. Data were collected between November 2018 and August 2020. Between March and August 2020, changes to health service procedures and protocols due to COVID‐19 meant that some sensory tools were not able to be used for interventions. All participants of the study were assessed for sensory processing (A/ASP), cognitive functioning (ACLS), occupational functioning (COPM), health and social functioning (HoNOS), and levels of distress (ERS). The participants were then followed up for a period of up to 6 months where the ACLS, COPM, and HoNOS were readministered at least 3‐monthly and the A/ASP 6‐monthly. ERS was administered to those participants using SM before and after each session.

### 2.5 Data analysis

Descriptive statistics were reported as mean (*SD*) for normally distributed continuous variables or median (*IQR*) for skewed variables. Categorical variables were summarised using frequencies and percentages. Differences in continuous variables at baseline between the control and intervention groups were tested with independent groups *t*‐tests, substituted with Mann–Whitney *U* tests for variables that were not normally distributed. Differences in proportions between the groups were tested using chi‐square test or Fisher's exact test. Sensory processing patterns on the four domains of the A/ASP in the current participants were compared with those of adults without schizophrenia from the same geographic area in Queensland, Australia (Machingura et al., 2019), using independent *t*‐tests to determine if our study sample differed with the general population.

Data analysis on the outcomes was conducted using a series of planned comparisons including independent and paired *t‐*tests. A mixed model ANOVA was used whenever statistically appropriate. Paired samples *t*‐tests were used to compare the mean pre‐test and post‐test scores across a range of functional outcomes (distress, occupational functioning, clinician‐rated functioning, and cognitive functioning). Independent *t*‐tests were used to test for differences in the change of scores on outcome measures between intervention and control participants. A 2 × 2 mixed model ANOVA was undertaken to investigate the impact of SM on the behaviour, impairment, symptoms, and social functioning of patients with schizophrenia using HoNOS Aggregate scores. The method of using HoNOS Aggregate scores was regarded appropriate as a similar method of using HoNOS Total scores was previously found to be valid (Green et al., 2007).

In both the participant and comparison groups, data were missing at random on the distress, cognitive functioning, occupational functioning, health, and social variables. To optimise power in our limited sample before conducting inferential tests, we applied multiple imputation based on 40 imputed datasets to account for missing values (38%). Multiple imputation restores the natural variability of the missing data by predicting missing data using correlated existing data from other variables, repeatedly in multiple imputed datasets. This method produces appropriate results in the presence of a small sample size or a high number of missing data (Kang, 2013). All analyses were performed with SPSS Version 26, and *p* < .05 was deemed to be statistically significant.

## 3 RESULTS

### 3.1 Participants

The intervention group (*n* = 30) and control group (*n* = 11) did not differ significantly in age, gender, education, or the number of admissions (see Table 1). Due to the presence of many low cell counts, pairwise comparisons were tested for each phase of treatment, and we found differences in phases of treatment such that the control group included a higher proportion of participants in acute treatment, whereas the intervention group had a higher proportion of participants in intensive extended treatment phase. This difference was considered when interpreting the findings as a potential bias.

**TABLE 1:** Demographic characteristics of study participant groups

	Control group (*n* = 11)	Intervention group (*n* = 30)	*p*‐value
Age (years), mean (SD)	42.7 (7.4)	36.4 (10.8)	.08
Gender			.73
Male	4 (36.4)	14 (46.7)	
Female	7 (63.6)	16 (53.3)	
Education			.65
Non‐tertiary	9 (81.8)	26 (86.7)	
Tertiary	2 (18.2)	4 (13.3)	
Admissions, median (IQR)	3 (3–8)	3 (3–4)	.77
Phase of treatment^a^			
Assessment only	0 (.0)	1 (3.3)	
Acute^b^	8 (72.7)	7 (23.3)	
Intensive extended^b^	0 (.0)	15 (50.0)	
Functional gain	1 (9.1)	2 (6.7)	
Consolidating gain	2 (18.2)	5 (16.7)	

*Note*: Data are reported as *n* (%) unless otherwise specified.

^a^ Due to low counts in many of the cells, only pairwise comparisons were tested for significance within each treatment phase.

^b^ The proportions between groups were significantly different (*p* < .05).

### 3.2 Sensory processing for people with schizophrenia

We compared sensory processing patterns of those participants with schizophrenia and those of the general population from the same geographical region. Although most participants were of the same age range, participants with schizophrenia were slightly younger, with a mean age of 36.4 years (*SD* = 10.8), whereas the comparison group had a mean age of 42.3 years (*SD* = 18.5). In terms of ethnicity, all participants with schizophrenia were Caucasians compared with 63% Caucasians in the comparative group. Participants with schizophrenia had higher scores in Low Registration and Sensation Avoiding than those of the general population. The results are presented in Table 2, alongside the range of normative scores for Adult and Adolescent Sensory Profile.

**TABLE 2:** Comparison of sensory processing in adults with and without schizophrenia

	Schizophrenia diagnosis (*n* = 41)	No schizophrenia diagnosis^a^ (*n* = 80)	Mean difference (95% CI)	*p*‐value	Normative mean scores^b^ (most people)
Low Registration	36.7 (8.5)	31.6 (7.6)	−5.16 (−8.40–1.90)	.01*	24–35
Sensation Seeking	48.3 (7.2)	47.9 (7.6)	−.38 (−3.49, 2.74)	.81	43–56
Sensory Sensitivity	37.2 (7.1)	34.6 (7.3)	−2.60 (−5.59, .40)	.09	26–41
Sensation Avoiding	42.0 (9.5)	36.5 (8.4)	−5.52 (−9.1, −1.88)	.01*	27–41

*Note*: Summary statistics are reported as mean (SD) for adults with schizophrenia and healthy adults.

^a^ Machingura et al. (2019) study data.

^b^ Brown et al. (2001) study norms.

* Statistically significant at *p* < .05.

### 3.3 Comparison of outcomes within intervention group

Paired samples *t*‐tests were used to compare intervention group participants' pre‐ and post‐test scores on the outcome measures (Table 3) There were statistically significant improvements between start and end scores for self‐rated distress, *t*(29) = −4.35, *p* < .001, and distress as rated by staff, *t*(29) = −4.03, *p* = <.001. There was also statistically significant improvements between start and end scores for health and social functioning as measured by HoNOS, *t*(29) = 4.68, *p <* .001. Results also showed statistically significant differences in occupational performance and no statistical significance in occupational satisfaction. No statistical difference was obtained between pre‐and post‐test scores for sensory processing scores and cognitive functioning measures. When the original data were analysed, we found the same pattern of statistically significant changes as for the imputed data.

**TABLE 3:** Change in functional outcomes within the intervention group

Measure	Start mean (SE)	End mean (SE)	Mean difference (95% CI)	*t‐*value	*Effect size Cohen's d*	*p*‐value
Occupational Performance	4.16 (.53)	5.52 (.72)	−1.37 (−2.72, −. 18)	−2.01	1.32	.047*
Occupational Satisfaction	3.66 (.56)	5.45 (.90)	−1.79 (−3.59, .01)	−1.97	.92	.052
Cognitive Functioning	5.03 (.11)	5.03 (.10)	−.01 (−.09, .08)	−.18		.854
Distress Levels—Consumer Rating	4.76 (.42)	6.69 (.41)	−1.93 (−2.80, −‐1.05)	−4.35	1.14	<.001*
Distress Levels—Staff Rating	5.35(.49)	7.12 (.44)	−1.75 (−2.61, −.90)	−4.03	.89	<.001*
HoNOS Aggregate	4.40 (.47)	2.52 (.38)	1.88 (1.09, 2.66)	4.68	.72	<.001*
*Sensory processing*						
Low registration	36.13 (1.64)	36.09 (1.81)	.03 (−1.46, 1.52)	.04		.966
Sensation Seeking	48.21 (1.39)	47.91 (1.37)	.29 (−.73, 1.31)	.55		.580
Sensory Sensitivity	37.26 (1.32)	37.49 (1.39)	−.23 (−1.90, 1.44)	−.27		.789
Sensation Avoiding	42.18 (1.77)	42.16 (1.72)	.02 (−2.05, 2.07)	.02		.988

*Notes*: Cohen's d effect size interpretation: .2 small, .5 medium, .8 large. Positive effect sizes represent improvement on the measure **Scores: Distress**: Higher score means consumer feeling calmer/relaxed. **Occupational Performance and Satisfaction**: Higher score means consumer performing and functioning better. **HoNOS**: Lower score means consumer has less symptoms and is feeling better.

* The start and end scores were significantly different (*p* < .05).

### 3.4 Comparisons of outcomes change scores between groups

Independent *t*‐tests were used to compare change score measures between the intervention and control groups. The results indicated that there were no statistically significant differences across all outcomes between the 30 participants who used SM compared with the 11 participants in the control group. The patterns seen in original data did not change following multiple imputations. Detailed results are presented in Table 4.

**TABLE 4:** Comparison of change in functional outcomes between the control and intervention groups

Variable	Change in control mean (SE)	Change in intervention mean (SE)	Mean difference (95% CI)	*p*‐value
Occupational Performance	−1.23 (1.66)	−1.37 (.68)	.14 (−3.72, 3.99)	.944
Occupational Satisfaction	−1.61 (1.89)	−1.79 (.91)	.18 (−4.55, 4.91)	.940
Cognitive Functioning	−.15 (.13)	−.01 (.2)	−.14 (−.41, .13)	.297
Distress Levels—Consumer Rating	−1.57 (1.13)	−1.93 (.44)	.36 (−1.94, 2.66)	.756
Distress Levels—Staff Rating	−2.00 (.58)	−2.00 (.41)	.01 (−1.38, 1.39)	.996
HoNOS Aggregate	2.40 (1.07)	1.88 (.40)	.52 (−1.74, 2.78)	.650
*Sensory processing patterns*				
Low Registration	1.64 (2.91)	.04 (.76)	1.60 (−4.30, 7.51)	.590
Sensation Seeking	1.95 (1.75)	.29 (.52)	1.66 (−1.95, 5.26)	.364
Sensory Sensitivity	4.37 (3.21)	−.23 (.85)	4.60 (−1.98, 11.17)	.168
Sensation Avoiding	4.27 (3.52)	.02 (1.05)	4.25 (−2.71, 11.20)	.229

Similar results were also found using a mixed model ANOVA where a main effect for time was found for health and social functioning, *F*(3.87) = 21.50, *p* ≤ .001, partial $\eta^{2}$ = .36, with staff reporting more health and social functioning problems for patients before the intervention (*M* = 3.76, *SD* = 3.02) than after the intervention (*M* = 2.00, *SD* = 2.10). However, a main effect of group was not significant, *F*(0.51) = .04, *p* = .84, partial $\eta^{2}$ = .01, *p* = .51. There was no significant Group × Time interaction.

## 4 DISCUSSION

The literature suggests that the goal of treatment in people with schizophrenia is to improve their social and occupational functioning (Rahman & Lauriello, 2016). This study sought to explore the effectiveness of SM. This knowledge is vital for practitioners to be able to prescribe, utilise, recommend, or guide practice. The hypothesis was that SM interventions would lead to greater improvements in reported occupational, health, and social outcomes for people with schizophrenia than treatment approaches that did not include SM interventions. Findings from this study suggest that SM interventions may not be associated with better outcomes than current interventions but instead may complement them. Overall, the results from this study show similar health, social, and occupational outcomes as is found with treatment as usual. There were no statistically significant differences found between groups, as participants with schizophrenia who received SM interventions in this study showed similar improvements on the outcome measures as those in the control group.

There were statistically significant pre–post improvements on scores in levels of self‐rated distress and on symptom relief and health and occupational functioning for those participants who had received SM. This finding suggests that patients experience benefits from both a user perspective, as reported in the self‐rated measure of distress and the self‐rated measure of occupational performance, and from a staff perspective, as measured by staff rated health and social functioning measures such as HoNOS. Comparisons with the control group cannot be made as the ERS was not administered in the control group due to the naturalistic approach used in this study. The results offer further evidence that SM interventions offer pre‐ to post‐intervention improvements across a number of variables including distress, health, social, and occupational functioning measures. These findings suggest that providing SM interventions to individuals with schizophrenia may offer distress relief as well as assist with improving their satisfaction with performance in daily life. Importantly, it should be noted that the same improvements were found among people with schizophrenia who were initially considered to be eligible for SM interventions but who instead received other forms of treatment.

The findings of this study suggest that potential benefits of SM interventions to people with schizophrenia appear to be immediate to short term, for example, decreased distress levels and improved health and social functioning within the past 2 weeks. Similar findings of improvement after using SM interventions have been reported in previous studies (Lloyd et al., 2014; Yakov et al., 2018). Findings from previous studies were based on heterogenous groups of participants, and this study adds the understanding of SM interventions when used on people with schizophrenia more specifically.

Findings on self‐perceived occupational functioning were statistically significant. Despite this self‐reported improvement in performance, it is not possible to attribute the finding solely to SM interventions as their use also implies a more active daily life, which may contribute to improvement in perception of daily functioning (Rahman & Lauriello, 2016). Additionally, similar improvements were seen in the control group; therefore, this result cannot be attributed to SM interventions. The results of this study suggest that SM interventions did not impact on the global cognitive processing abilities of participants. This finding adds to the view that these interventions offer immediate to short‐term relief to users and improve occupational performance within one's environment rather than changes to global cognitive performance (Bar‐Shalita & Cermak, 2016).

This study considered whether people with schizophrenia's sensory profiles were atypical when compared with the general population (Machingura et al., 2019). In this study, statistically significant differences in Low Registration and Sensation Avoiding were found between participants who had schizophrenia and healthy participants we had recruited in an earlier study. Participants with schizophrenia had higher scores than those in the healthy participants study and higher scores than most people. Findings of this study suggest that participants with schizophrenia have a higher neurological threshold than most people and may require more intense stimuli than most people for them to be able to register it. Similarly, previous studies also found that participants with schizophrenia had higher scores than participants without schizophrenia participants (Brown et al., 2002; Brown et al., 2020; Machingura et al., 2019). This implies that they are less likely to take measures to avoid sensory stimuli that they found to be unpleasant to them.

### 4.1 Implications for practice

Although the study did not find statistically significant differences between the control and intervention groups, it is possible that the intervention had advantages that were not discerned with the current study design and measures. There were improvements on perceived occupational functioning and a reduction in distress found within the intervention group, which suggests that SM interventions might be helpful in the milieu of interventions offered to people with schizophrenia as a complementary intervention. The lack of statistical significance between the intervention and control groups implies that a cautious approach where the state of the evidence is explained to patients and potential users prior to involvement is needed. If practitioners choose to implement this approach clinically, then they should carefully document intervention content, context, client responses to the treatment, and changes in client functioning (or occupational participation and engagement).

The results of this study support previous findings that suggest that people with schizophrenia process sensory information differently to other people. Findings of this study suggest that participants with schizophrenia were more likely to miss sensory information and are less likely to initiate avoidance of adverse sensory stimuli. Sensory processing considerations should therefore be made when providing services to people with schizophrenia. These atypical sensory processing patterns, however, did not change within the 6 months' time measured, suggesting that they may be difficult to change or there is need to develop more sensitive measurement tools for sensory patterns. The clinical implications may be that frequent assessments may not be necessary if using the A/ASP. This finding may save clinicians and researchers valuable time if considered.

### 4.2 Limitations

There were several limitations to this study. Firstly, the training package used still requires further validation as that could have affected the knowledge and skill level of staff providing interventions. Secondly, the timing of reassessments could not be standardised as individuals were admitted and discharged at different times. Thirdly, there were differences in phases of treatment, with the control group having a higher proportion of participants in acute treatment, whereas the intervention group had a higher proportion of participants in intensive extended treatment phase. Furthermore, participants were not randomly allocated to the intervention and control groups. This was a practical, ethical decision not to exclude some participants from the intervention who could otherwise benefit from it. Instead, a naturalistic approach was used, which could have biased the findings. The effects of using active controls receiving other psychosocial interventions and the imbalance between numbers of participants between groups however could have contributed to the lack of statistically significant difference between groups. We also had a relatively small sample because of the impact of COVID‐19 pandemic, which made recruitment and data collection difficult due to government restrictions being implemented at the time of data collection. Also, the participants were in varying phases of treatment, which meant that some potential gains in some participants might have been minimised because they were more unwell compared with others at the time. Furthermore, the follow‐up period varied for practical reasons, which meant that some participants had simply not had enough time to realise the effects of SM interventions more fully. We also used self‐reported measures to measure sensory processing and cognitive and occupational functioning, which could have created biases related to memory, cognition, and insight. These difficulties are common in people with schizophrenia (Brown & Dunn, 2002). The sensory processing measure used in this study does not measure the intensity but rather the frequency of the response. The intensity of distress caused by stimuli might be much greater for people with schizophrenia even though frequency might not be as different (Brown et al., 2002). Another factor to be considered is the possibility of lack of sensitivity of the measures used not specifically designed to measure outcomes of SM interventions. The relatively short follow‐up period, missing data, and the small control group size reduce the generalisability of the results.

### 4.3 Suggestions for further studies

Further randomised controlled trials with larger samples and longitudinal studies to measure occupational performance over time using more sensitive measures of cognitive and occupational functioning are recommended. The findings from this study also suggest that current measures might not have captured the effectiveness of SM interventions; therefore, alternative measures possibly including individually relevant measures (e.g. degree of goal attainment) are needed. Further evaluation and standardisation of SM interventions training would also aid understanding of effectiveness of SM interventions.

## 5 CONCLUSION

This study has built on the current understanding on the effectiveness of SM interventions for people with schizophrenia. Findings suggest potential distress relief as well as improved health, social, and occupational performance in daily activities. Furthermore, this research found that people with schizophrenia in this study had sensory processing patterns that were different to those of the general population. The sensory profiles did not change in the 6 months participants were enrolled in the study. The study findings suggest that further research is needed to further test these findings with larger samples and over longer periods. A randomised controlled trial would be the ideal design to provide evidence on efficacy of SM interventions. Investigating new and more sensitive tools for assessing sensory processing patterns as well as developing and standardising training packages would also be beneficial.

## CONFLICT OF INTEREST

The authors know of no conflicts of interest with this publication, and there has not been any significant financial support for this work that could influence this publication.

## AUTHOR CONTRIBUTIONS

TM: Scoping the research, synthesising the literature, project design, the collection of some of the data, data analysis, interpreting data, writing the first draft, and editing the manuscript. HG, KM, CL, and DS: Scoping the research, project design, interpreting data, and editing the manuscript. ER: Scoping the data analysis, data analysis editing, and interpretation.

## ETHICS STATEMENT

The study commenced after receiving ethical clearance from Gold Coast University Hospital Human Ethics Review Committee (HREC/17/QGC/151) and Griffith University Human Research Ethics Committee (GU: 2019/079).

## Data Availability

The data that support the findings of this study are available on request from the corresponding author [TM]. The data are not publicly available due to their containing information that could compromise the privacy of research participants.
